# Supplementary material for: Association between oral microbiome and seven types of cancers in East Asian population: a two-sample Mendelian randomization analysis
Source: Front Mol Biosci. 2023 Nov 21;10:1327893. doi: 10.3389/fmolb.2023.1327893 (PMC10702768; doi:10.3389/fmolb.2023.1327893)
Supplement: Supplementary file 1 [file DataSheet1.ZIP › Supplementary Materials/MR plots of saliva/saliva═╝/colorectal cancer/pheno.907_to_colorectal cancer_funnel.pdf]

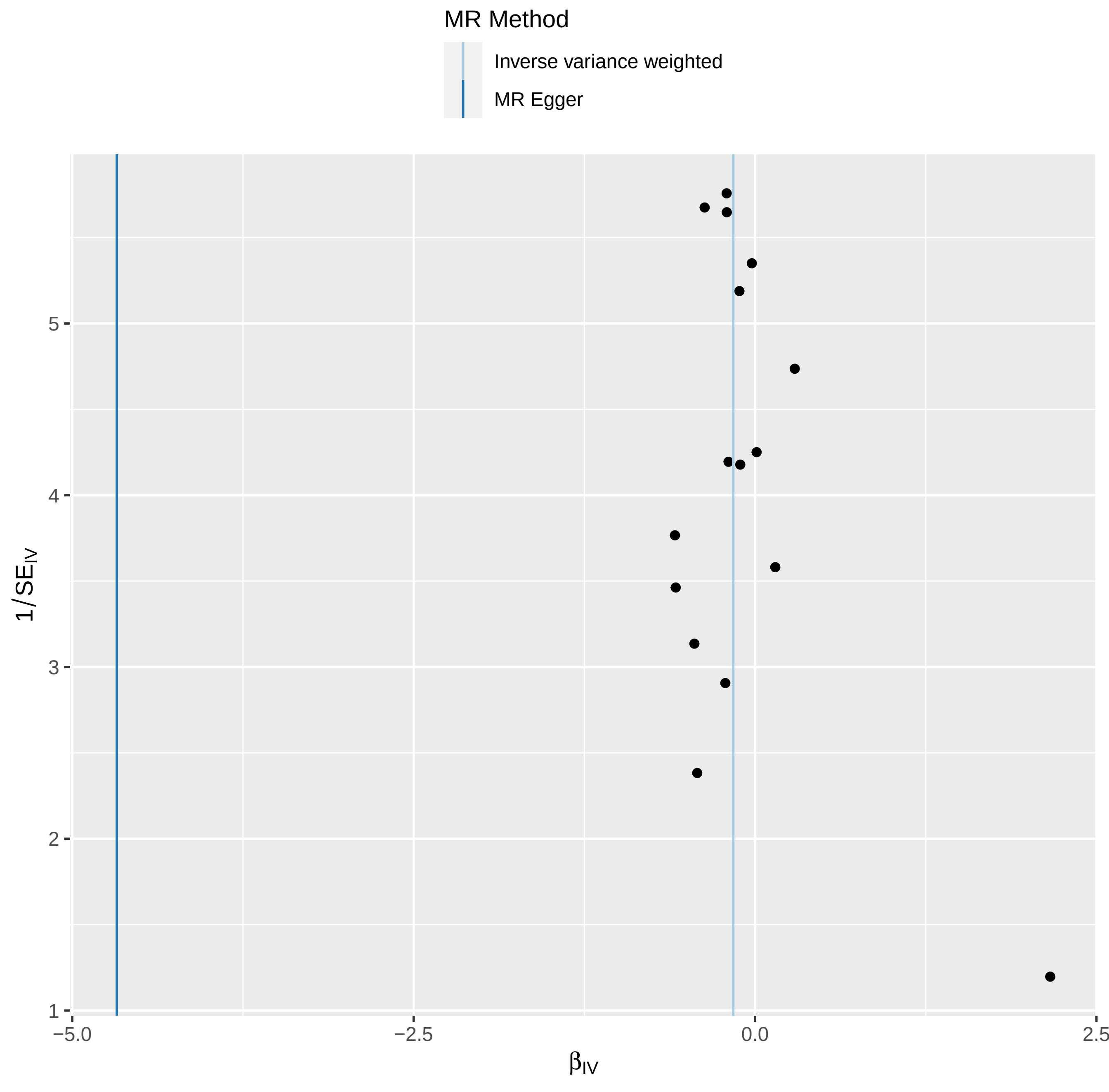

MR Method
Inverse variance weighted
MR Egger
1/SE_IV
5
4
3
2
1
−5.0
−2.5
0.0
2.5
β_IV
